# Association of Necrotizing Wounds Colonized by Maggots with *Ignatzschineria*–Associated Septicemia

**DOI:** 10.3201/eid2110.150748

**Published:** 2015-10

**Authors:** Cécile Le Brun, Martin Gombert, Sylvie Robert, Emmanuelle Mercier, Philippe Lanotte

**Affiliations:** Centre Hospitalier Régional Universitaire de Tours, Tours, France (C. Le Brun, M. Gombert, S. Robert, E. Mercier, P. Lanotte);; Université François Rabelais de Tours, Tours (S. Robert, P. Lanotte);; Institut National de la Recherche Agronomique, Nouzilly, France (S. Robert, P. Lanotte)

**Keywords:** Ignatzschineria, Ignatzschineria ureiclastica, Wohlfahrtia, bacteremia, fly maggots, bacteria, parasites, wounds

**To the Editor:** *Ignatzschineria* is a recently described genus of bacteria that have been rarely implicated in human disease (*1*–*3*). We report a patient in France with septicemia caused by *I. ureiclastica*.

In October 2013, a 69-year-old man was found unconscious in a forest close to Tours in the Loire Valley, France. The patient had hypotension with auricular fibrillation complicated by cardiorespiratory arrest and was admitted to the general intensive care unit of Tours University Hospital. He also had cyanosis of the extremities, a necrotic skin lesion on the right shoulder, and a large number of maggots around the genital organs. Empiric treatment with ceftriaxone was initiated. Blood cultures on admission revealed several microbes: *Enterococcus faecalis*, *Enterobacter cloacae*, *Providencia stuartii*, *Corynebacterium* spp., and a gram-negative bacillus resembling *Pseudomonas*. This bacillus was sensitive to all β-lactams, aminosides, fluoroquinolones, colistin, and trimethoprim/sulfamethoxazole but was resistant to fosfomycin. Ten days after admission to the hospital, the patient was found dead in his bed from no evident cause, despite recent improvement of his clinical state. No autopsy was conducted.

The unidentified gram-negative bacillus was an oxidase-positive strict aerobe. The 16S rRNA and *gyrB* genes were amplified and sequenced (*4*,*5*). The 897-bp 16S rRNA sequence obtained for the bacterium was 99% identical to sequences from *I. larvae* type strain L1/68T (GenBank accession no. AJ252143) and *I. ureiclastica* type strain FFA3T (GenBank accession no. EU008089). The 973-bp *gyrB* sequence of the isolate was 96% similar to the sequence of *I. ureiclastica* type strain FFA3T (GenBank accession no. FJ966120) and 92% with *I. larvae* type strain L1/68T (GenBank accession no. FJ966121). The 16S rRNA and *gyrB* sequences (GenBank accession nos. KR184134 and KR184135) were compared with those of all members of the genus *Ignatzschineria* and with those of several species belonging to the class Gammaproteobacteria. Two phylogenetic trees were deduced by the neighbor-joining method (Figure).

**Figure:**
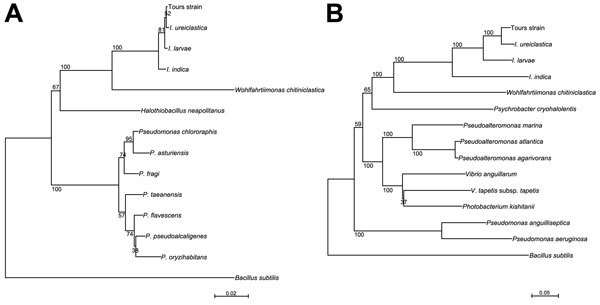
Phylogenetic trees showing relationships between the clinical isolate identified in this study (“Tours strain”) and type strains of members of the genus *Ignatzschineria*. A) Relationships among 16S rRNA sequences of “Tours strain” (GenBank accession no. KR184134) and *Ignatzschineria* strains; scale bar represents 2% differences in nucleotide sequence. B) Relationships among *gyrB* sequences of “Tours strain” (GenBank accession no. KR184135) and *Ignatzschineria* strains; scale bars represent 5% differences in nucleotide sequence. *Bacillus subtilis* was included as an outgroup organism. Numbers at branch nodes are bootstrap values.

The genus *Ignatzschineria*, which is the revised nomenclature for *Schineria*, was first described in 2001. It comprises 3 species: *I. larvae*, *I. indica*, and *I. ureiclastica* (*6*–*8*), and belongs to the family *Xanthomonadaceae*, class Gammaproteobacteria,. All 3 species have been isolated from larvae *Wohlfahrtia magnifica* flies (*9*), which are found in Europe, Asia, and North Africa and cause myiasis in several animal species but rarely in humans. *Ignatzschineria* spp. is the dominant species in the anterior portion of the digestive tract in larvae, together with *Providencia* (*9*). *Providencia* was also found in blood cultures from this patient. Cases of *I. larvae* and *Ignatzschineria* sp. bacteremia were reported in France: 1 in a homeless patient (*2*) and the other in a patient with type 2 diabetes (*1*), both with a foot wound invaded by maggots. Three cases of *I. indica* infection were recently described in the United States: 2 cases of bacteremia and 1 urinary tract infection (*3*). These 3 cases were clearly associated with fly larvae infestations and myiasis.

The presence of *I. ureiclastica* in the blood cultures of the patient reported here and the presence of bacteria from the same genus in 4 other cases of bacteremia suggest an association between *Ignatzschineria* bacteremia and wounds infected by maggots in patients with poor hygiene. Systematic blood cultures should therefore be conducted for such patients. The epidemiologic importance of *Ignatzschineria* spp. might have been underestimated because of the presence of other microbes in samples and identification difficulties, which in some cases might have led to a conclusion of simple contamination.

The species of fly larvae found in wounds and the bacteria transmitted appear to differ among geographic regions. In France, *I. larvae* and *I. ureiclastica* are the species associated with the *W. magnifica* fly, which is present in Europe, Asia, and North America. In the United States, the 3 human infections reported were all caused by *I. indica* and seemed to be associated with larvae of the *Phaenicia sericata* fly, found throughout the world. A geographic specificity of *Ignatzschineria* spp. linked to the geographic distribution of fly larvae is therefore remarkable.

The larvae used in maggot therapy are “sterile” larvae of the *P. sericata* fly. A possible risk for infection with *Ignatzschineria* exists with larval therapy, especially with *I. indica*.

The pathogenic power of *Ignatzschineria* spp. remains to be demonstrated. However, a wound invaded by maggots seems to be strongly associated with the presence of *Ignatzschineria* spp. in clinical samples, with the possibility of a specific geographic distribution of the species implicated. Clinicians and microbiologists should be aware of the possibility of invasive *Ignatzschineria* infections in presence of maggots in patients with poor hygiene and should check specifically for this bacterium.

## References

[1] Maurin M, Delbano JN, Mackaya L, Colomb H, Guier C, Mandjee A, Human infection with *Schineria larvae.* Emerg Infect Dis. 2007;13:671–3. 10.3201/eid1304.061151 17561570 PMC2725969

[2] Roudiere L, Jean-Pierre H, Comte C, Zorgniotti I, Marchandin H, Jumas-Bilak E. Isolation of *Schineria* sp. from a man. Emerg Infect Dis. 2007;13:659–61. 10.3201/eid1304.061255 17561571 PMC2725973

[3] Barker HS, Snyder JW, Hicks AB, Yanoviak SP, Southern P, Dhakal BK, First case reports of *Ignatzschineria* (*Schineria*) *indica* associated with myiasis. J Clin Microbiol. 2014;52:4432–4. 10.1128/JCM.02183-14 25297331 PMC4313336

[4] Rådström P, Bäckman A, Qian N, Kragsbjerg P, Påhlson C, Olcén P. Detection of bacterial DNA in cerebrospinal fluid by an assay for simultaneous detection of *Neisseria meningitidis, Haemophilus influenzae*, and streptococci using a seminested PCR Strategy. J Clin Microbiol. 1994;32:2738–44 . 7852565 10.1128/jcm.32.11.2738-2744.1994 PMC264152

[5] Yamamoto S, Harayama S. PCR amplification and direct sequencing of *gyrB* genes with universal primers and their application to the detection and taxonomic analysis of *Pseudomonas putida* strains. Appl Environ Microbiol. 1995;61:1104–9 . 7793912 10.1128/aem.61.3.1104-1109.1995 PMC167365

[6] Tóth E, Kovács G, Schumann P, Kovács AL, Steiner U, Halbritter A, *Schineria larvae* gen. nov., sp. nov., isolated from the 1st and 2nd larval stage of *Wohlfahrtia magnifica* (Diptera: Sarcophagidae). Int J Syst Evol Microbiol. 2001;51:401–7 . 10.1099/00207713-51-2-401 11321085

[7] Tóth EM, Borsodi AK, Euzéby JP, Tindall BJ, Márialigeti K. Proposal to replace the illegitimate genus name Schineria Tóth et al. 2001 with the genus name *Ignatzschineria* gen. nov. and to replace the illegitimate combination *Schineria larvae* Tóth et al. 2001 with *Ignatzschineria larvae* comb. nov. Int J Syst Evol Microbiol. 2007;57:179–80. 10.1099/ijs.0.64686-0 17220462

[8] Gupta AK, Dharne MS, Rangrez AY, Verma P, Ghate HV, Rohde M, *Ignatzschineria indica* sp. nov. and *Ignatzschineria ureiclastica* sp. nov., isolated from adult flesh flies (Diptera: Sarcophagidae). Int J Syst Evol Microbiol. 2011;61:1360–9. 10.1099/ijs.0.018622-0 20584814

[9] Tóth EM, Hell É, Kovács G, Borsodi AK, Márialigeti K. Bacteria isolated from the different developmental stages and larval organs of the obligate parasitic fly, *Wohlfahrtia magnific*a (Diptera: Sarcophagidae). Microb Ecol. 2006;51:13–21. 10.1007/s00248-005-0090-6 16382282

